# Perspectives of health professionals on the best care settings for pediatric trauma casualties: a qualitative study

**DOI:** 10.1186/s13584-018-0207-2

**Published:** 2018-03-28

**Authors:** Raya Madar, Bruria Adini, David Greenberg, Yehezkel Waisman, Avishay Goldberg

**Affiliations:** 1 Pediatric Surgery Department, Soroka University Medical Center, Faculty of Health Sciences, Ben-Gurion University of the Negev, Beer-Sheva, Israel; 2 Department of Disaster Management and Injury Prevention, School of Public Health, Sackler Faculty of Medicine, Tel-Aviv University, Tel-Aviv, Israel; 3 Pediatric Infectious Disease Unit, Pediatrics Department, Soroka University Medical Center, Faculty of Health Sciences, Ben-Gurion University of the Negev, Beer-Sheva, Israel; 4 Department of Emergency Medicine, Schneider Children’s Medical Center of Israel, Petah Tikva, Israel; 5 School of Continuing Medical Education, Sackler Faculty of Medicine, Tel-Aviv University, Tel-Aviv, Israel; 6 Department of Health Systems Management, Faculty of Health Sciences, Ben-Gurion University of the Negev, Beer Sheba, Israel; 7 PREPARED Center for Emergency Response Research, Ben-Gurion University of the Negev, Beer Sheba, Israel

**Keywords:** Emergency services, Trauma, Pediatrics, Health policy

## Abstract

**Background:**

Critically-injured children are frequently treated by providers who lack specialty pediatric training in facilities that have not been modified for the care of children. We set out to understand the attitudes and perspectives of policy makers, and senior nursing and medical managers in the Israeli healthcare system, concerning the provision of medical care to pediatric trauma casualties in emergency departments.

**Methods:**

We conducted semi-structured interviews with 17 health professionals from medical centers across Israel and the Ministry of Health. The interviews were analyzed by qualitative methods.

**Results:**

There was lack of clarity and uniformity concerning the definition of a pediatric trauma casualty. All of the participants attributed extreme importance to the professional level of the care team manager, and most suggested that this should be a pediatric emergency medicine specialist. They emphasized the importance of around-the-clock availability of pediatric medical teams to care for young trauma casualties, and the crucial need for caregivers to be equipped with a wide variety of professional skills for the adequate treatment of a broad spectrum of injuries. All participants described significant variability in pediatric-care training and experience among physicians and nurses working in emergency departments. Most participants believe that pediatric trauma casualties should be treated in designated pediatric emergency departments, in a limited number of medical centers across the country.

**Conclusions:**

Our findings indicate that specialized pediatric EDs would constitute the best location for intake of children with major traumatic injuries. Pediatric emergency medicine specialists should manage trauma cases using pediatric surgeons as ad-hoc consultants. The term ‘pediatric patient’ should be defined to allow trauma patients to be referred to the most appropriate ED. Teams working at these EDs should undergo specialized pediatric emergency medicine training. Finally, to regulate the key aspects of trauma care, clear statutory guidelines should be formulated at national and local levels.

## Background

Traumatic injury refers to physical injuries of sudden onset and severity which require immediate medical attention. Children are injured in a wide variety of geographic locations, and the involvement of local and regional centers is paramount to optimizing care for pediatric casualties [1]. The structure and care processes for critically-injured children vary [2]. In the USA, nearly 90% of children are seen in general emergency departments (EDs) [3] and many of them are treated in level I or level II adult trauma centers [4]. The majority of clinicians of all disciplines are understandably anxious when faced with a child presenting to a general ED [5]. Frequently, these children are treated by providers who lack specialty pediatric training in facilities that have not been modified for the care of children [6]. These factors impede the quality of care; it has been shown that younger and more seriously-injured children have better outcomes when treated at trauma centers located within children’s hospitals or at trauma centers that integrate pediatric and adult trauma services [1, 7, 8]. In Israel, there are 6 level I trauma centers and 14 regional trauma centers [9]; however there are no pediatric trauma centers and most injured children are treated in general EDs [10].

We examined the attitudes of policy makers, senior medical and nursing managers in the Israeli healthcare system, concerning the provision of medical care to pediatric trauma casualties in EDs.

## Methods

### Study design and interview guide

We chose a qualitative methodology for the study as it is known to enable flexibility and openness to a variety of possible answers, and is uniquely suited for identification of previously unknown processes, assessment of their effects and elucidation of underlying motivations of healthcare providers [11]. A semi-structured interview guide was constructed by the study team on the basis of current literature. The interview topics are shown in Table 1.

**Table 1:** Interview guide

• Please tell me about your professional experience.
• In what ways is treatment of children after multiple casualty incidents and trauma in Israel distinct?
• What are the challenges of treating such children?
• How should the various care givers collaborate when treating children after multiple casualty incidents and trauma?
• When it comes to treating children after multiple casualty incidents and trauma, what is your opinion on the skills of the emergency department teams in each type of emergency department (general/pediatric)?
• What are the skills and resources required for admission and treatment of children after multiple casualty incidents and trauma? This could include technological resources, training and other elements you can think of.
• Are you aware of any regulatory mechanism that assesses the treatment of children after multiple casualty incidents and trauma? How should lessons be learned?
• According to the literature, only a few emergency departments in the USA are prepared according to leading pediatric organizations’ guidelines. What, in your opinion, is the situation in Israel? What is its direction?
• What are the advantages and disadvantages of caring for children in each type of the emergency departments (general/pediatric)?
• In your opinion, which emergency department would be the most appropriate for treating children after multiple casualty incidents and trauma? Why? Does the severity of the casualty have any effect on choosing the emergency department? Do you think that caring for severe pediatric trauma casualties should only be done at specific institutions?
• In Israel there are several models for admission of pediatric trauma casualties. In the USA there are pediatric nurse practitioners who are in charge of admission of pediatric trauma casualties in the emergency department. Based on your experience, which models are you aware of? Would you recommend a specific model?
• How would changing the model of admission and care of children after multiple casualty incidents and trauma affect the position of the medical and nursing teams? How would it change the treatment-associated economic expenditures?
• Do you foresee any other effects?
• In 2008 the Ministry of Health and the Israeli Association for Emergency Medicine recognized pediatric emergency medicine as a medical subspecialty for residency. What is your position on this development in pediatric healthcare? How does it affect the treatment frame/setting?
• Another ongoing change is that pediatric surgery is turning into a specialty of its own. Do you think it has an effect on the location of admission into care and treatment? I would like to mention that there is evidence in the literature that this specialty is no longer associated with pediatric injury. What is your opinion on this issue?
• How do you foresee the effect of establishing pediatric hospitals within general hospitals on caring for injured children?
• From your experience, are there any additional issues that would like to mention in regards to this study?

### Participants

Purposive sampling was used to obtain a sample of 17 participants with diverse characteristics and points of view that were likely to provide various approaches towards the subject under investigation. The participants work in the Israeli Ministry of Health and in medical centers across Israel. They are considered highly-experienced leaders in the field of pediatrics and trauma medicine (Table 2).

**Table 2:** Characteristics of the study population

Variables	Number of participants *N* = 17
Demographics	
Age range	40–65 years
Gender	
Males	14
Females	3
Occupation	
Trauma expert	2
Head of pediatric emergency medicine department	2
Head of general emergency medicine department	2
Director of children’s hospital	2
Director of general hospital	2
Senior physician (general and pediatric emergency medicine, pediatric surgery)	2
Senior nurses	2
Policy leaders	3

### Data collection

Interviews were conducted between February and May 2016. Sixteen interviewees provided consent to be recorded, and those interviews were recorded and transcribed from the recording; the single interview of a participant who refused to be recorded, was written down by the interviewer immediately following its completion. All interviewees provided consent for publication of the opinions expressed in the interviews. The study was reviewed and approved by the institutional ethics committee. Each participant signed an informed consent prior to participating in the study.

### Analysis

In the familiarization stage, interview transcripts were read meticulously with the general aim of identifying themes and common features. The preliminary in-depth review was followed by categorical analysis and classification of the text into text segments (“meaning units”). These segments served as a basis for creating a list of tags, representing a set of concepts conveyed in each interview. In a manner similar to that described by Gale et al., a thematic framework was developed based on pre-defined elements and themes that emerged from the familiarization stage.

The text was divided into 636 statements that comprised short sentences or longer texts that could not be further divided to understand their meaning. The statements were grouped into sub-categories and then into categories. The classification into categories was based on a pre-prepared list that was constructed following an extensive literature review [12]. Additional categories were added based on textual contents that were identified during the second reading. Category names were temporary and subject to change. The naming process was framed within the context of the question that was asked, and the data that was derived from the responses to it. Category names were modified at a later stage in order to preserve the original meanings, contexts and perceptions of the participant.

### Trustworthiness of the study

To strengthen the trustworthiness of analysis [13], several methods were used. Recording and transcription of interviews served to elevate the level of trustworthiness of the interviews. In addition, throughout the study, an investigator’s diary was kept, in which thoughts, feelings and subjective experiences that were raised during the study were listed, in order to minimize the potential effect of the investigator’s personal biography as a nursing manager of a pediatric surgical department. A colleague nurse from within the same pediatric surgical department reviewed the interviews and their respective analyses and assisted in preserving the original context as given in the interviews. The description of the field work utilized rich language, designed to convey the widest amount of information to allow the researchers to make an informed judgement as to the generalizability of the study. Last, the analyses were independently reviewed by the research team which included 3 professionals from the fields of medical disaster management, pediatric nursing and health systems management.

## Results

The key characteristics of the 17 senior healthcare stakeholders that were interviewed are presented in Table 2.

**Fig. 1:**
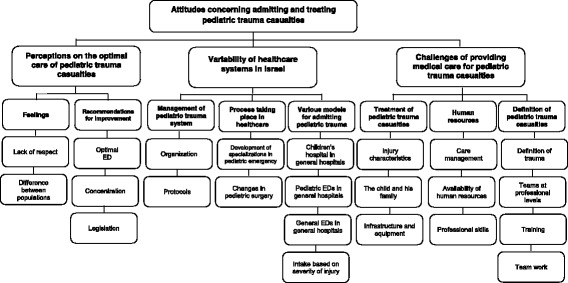
Category tree

We identified 3 major categories and 8 major sub-categories. These are summarized in Fig. 1 and described below. Representative quotes are provided in Table 3.

**Table 3:** Examples of quotes supporting the major categories and subcategories

Challenges in providing medical care to pediatric trauma casualties in emergency departments daily routine and during mass casualty events	
Subcategory	Examples of Quotes
Lack of uniformity concerning the definition of a child	“I have a problem with any definition of what a child is... anyone over 50 kilogramsor over the age of 14 years is an adult as far as I am concerned.” “In certain hospitals the age limit for the definition of a child was changed because of perceptions, and for all sorts of reasons not related to anything medical. Someone wanted to increase capacity at a certain place or decrease it elsewhere and then suddenly a [person is a] child until the age of 18.” “There are places in which [the definition of a child] is until the age of 14, there are places 16, however it suits, it depends.”
Medical Care management	“Sometimes, there is an ambiguity concerning who is responsible for managing the patient. Is it the (general) trauma surgeon, or the pediatric surgeon?” “Emergency medicine professionals do not understand anything about trauma.”
Availability of human resources	“Experts in pediatrics can treat an injured child correctly right from the start ... the approach of a specialist, no matter how qualified he is in treating adults, is completely different when required to treat an injured child” “The problem lies entirely within the small surgical professions, such as in neurosurgery, ophthalmology, ear nose and throat that we do not always, or not at all, have on call.”
Pediatric training and experience among physicians and nurses working in EDs.	“The problem is that a pediatric surgeon, by definition, almost never has training in pediatric trauma. The (general) trauma surgeon has experience in trauma but generally no experience with children.” “There may be a situation in which the majority of the medical teams have no previous experience in treating small children” “Who is assembling [training programs] for child care? Who is teaching trauma?” “I sent a nurse twice to an emergency medicine course but when I looked at the syllabus, I saw that the content she learnt was mostly irrelevant, so I felt that I was wasting the time of a very important resource [the nurse]”
Variability in the healthcare system (that affects) the provision of medical care to pediatric trauma casualties in EDs	
Subcategory	Examples of Quotes
Different models for admitting and treating pediatric trauma casualties in EDs	“We do all the primary care [in pediatric ED]. An injured child arrives, whether it is an orthopedic injury or a head injury. We, the pediatric ED doctors respond… perform all the tests, and we call in a surgeon at some stage… we deal with the neurosurgeons if needed”. “The existing mode of operation is, at least here in Israel, that a child with severe trauma is treated in the adult trauma room”.
The best setting for care of pediatric trauma casualties	
Subcategory	Examples of Quotes
The ED perceived as most suitable for pediatric casualties	“A child that is injured should be in a pediatric ED…similar to the way we treat a sick child, we should care for an injured child”.
Centralizing the treatment of injured children	“I think that [severe] pediatric trauma, that is a severe but rare disease, should be cared for in a centralized manner, in only a few medical centers.” “The advantage of a central pediatric trauma care structure, i.e. treatment in a limited number of centers, is that each center acquires more experience and thus improves its capacity for providing the specific care it is expected to deliver.”

*ED* Emergency department

### Category 1: Challenges in providing medical care to pediatric trauma casualties in EDs during daily routine and mass casualty events

We found a lack of clarity and uniformity concerning the definition of a pediatric trauma casualty. Some study participants defined children based on age or physical size, while others defined them according to sexual maturity or body weight. There were also those who related that economic considerations, such as the occupancy in certain hospital wards, were involved when defining a pediatric patient.

The human resource was repeatedly specified as the most vital component and as one of the key challenges in providing medical care to pediatric trauma casualties. This encompassed management of care, availability of manpower and level of professional skill.

All study participants attributed extreme importance to the professional level of the care team manager and emphasized the need for the manager to have the best skills for providing care to pediatric trauma casualties. The participants varied in their views regarding the preferable team-manager. According to most of our interviewees, this should be a pediatric emergency medicine specialist. Some participants perceived the pediatric surgeon as the preferred manager, but other participants stated that pediatric surgeons are usually not available for treating trauma cases in the ED (because they are mostly in the operating room). Some said that general surgeons are experienced in treating trauma casualties but do not have much experience in treating pediatric casualties. Study participants also differed in their views on the emergency medical teams best-suited to treat pediatric trauma casualties. On the one hand, pediatric ED teams were described as having the responsibility and appropriate skills for providing primary care to children. On the other hand, some of the participants were reserved with respect to the pediatric ED teams’ professional abilities and qualifications for treating trauma casualties.

Another challenge mentioned was the availability of caregivers. Study participants emphasized the importance of around-the-clock availability of pediatric medical teams to care for young trauma casualties. They described numerous situations in which there is a scarcity of pediatric trauma experts, as well as a shortage of all types of surgical teams: pediatric, orthopedic, plastic and ear nose and throat surgeons.

Participants described the crucial need for caregivers to be equipped with a wide variety of professional skills for the adequate treatment of a broad spectrum of injuries. These include, among others, skills in the areas of orthopedics, neurosurgery, sedation and imaging. In addition to professional skills, the medical teams should be able to express empathy and tolerance to attain the cooperation of the injured child as well as family members.

Differences of opinion were expressed regarding the best training for medical teams in charge of caring for pediatric trauma casualties. One participant stated that the current training level is inadequate, as there are no specific training programs focused on pediatric trauma casualties. Two study participants elaborated on the professional training they received in pediatric EDs in centers outside of Israel that included focus on the care of pediatric trauma casualties. As for the nursing staff, study participants stated that there are no programs for training nurses on the care of pediatric trauma casualties. Current nursing training programs either teach critical pediatric care, without specific focus on emergency care or alternatively, focus on emergency medicine with no regard for pediatric casualties.

### Category 2: Variability in the healthcare system affects the provision of medical care to pediatric trauma casualties in EDs

During the interviews, participants repeatedly noted the variability in admission and treatment of children in different Israeli medical centers. There are a number of designated institutions that care for children in Israel, only one of which is defined as an independent pediatric medical center. Most participants stated that due to the lack of available qualified personnel, the treatment of pediatric casualties with major trauma is extremely challenging in all of these pediatric medical facilities, including the independent pediatric medical center. Most frequently, pediatric trauma casualties are evacuated to general EDs and are often treated by trauma teams trained and experienced in treating adult casualties. Even at the pediatric medical center, severe pediatric trauma casualties are treated in the general ED at the adjacent Level I trauma center, and pediatric medical teams are dispatched to assist.

Two participants presented an alternative model for intake of pediatric trauma casualties, which is based on the severity of injury. In this model, children with mild trauma injuries are referred for admission and treatment in pediatric EDs, where care is managed by a team of pediatric ED physicians, while severe pediatric casualties are admitted to and treated in the general ED.

### Category 3: The best setting for care of pediatric trauma casualties

Various opinions were voiced with respect to the best suited ED for the care of pediatric trauma casualties. All participants felt that the most appropriate ED is the one that is best equipped to provide the best response. Most participants believe that pediatric trauma casualties should be treated in a designated pediatric ED, like other pediatric diseases. In a pediatric ED, the physical conditions are suitable for children and the staff is well-oriented and trained for providing care to children and their families. Some participants thought that intake into a pediatric ED should depend upon appropriate infrastructure and proximity to a general ED. A few participants thought that such decisions should be made independently at each medical institution.

Most of the participants favored the model of funneling severe pediatric trauma casualties to a limited number of medical centers across the country. Supportive statements stressed the large number of cases that will be treated at the designated centers, improving the skills of the medical teams and leading to better outcomes. A few arguments were raised against this paradigm: first, it could potentially impede the abilities of other hospitals to treat pediatric trauma casualties, which may become crucial in mass casualty events. This argument was contradicted by a study participant who claimed that medical teams, who are not skilled in trauma care under routine circumstances, will fail in emergencies. Second, it would add a lag time between the field and the ED for transport to the designated center and third, it would complicate the handling of families whose children are treated away from their homes. One participant suggested evacuating pediatric trauma casualties to the nearest medical center until stabilized, and then transferring them to a dedicated pediatric trauma center.

## Discussion

Pediatric patients are not small adults; their unique anatomy, physiology, and pathophysiology have to be considered in order to provide specialized care, especially during emergency situations. Their needs include practitioners trained in the care of children, specialized equipment, and appropriate resources for potential hospitalization and surgical intervention [14]. Previous studies related to the attitudes of healthcare professionals towards other aspects of pediatric emergency care, including family presence during resuscitation, or to the use of information technology in the ED [15]. To the best of our knowledge, this is the first study that assessed the perceptions and attitudes of healthcare professionals concerning the settings and resources required for providing optimal care to pediatric trauma casualties.

To provide optimal treatment to pediatric trauma casualties in the most appropriate setting and by the most suitable manager, the term “pediatric patient” should first be defined and should include the pediatric patient’s upper age cutoff. The definition of pediatric age varies greatly among hospitals in Israel due to various considerations [16]. The Israeli National Committee for the Management of Pediatric Multiple Casualty Incidents formed to improve the management of children involved in multiple casualty incidents, defined the upper age cutoff at 12 years [16]. There is also great variability in the definitions of pediatric patients in medical settings around the world. Some consider the upper age limit of pediatric patients as early as 12 years [2], some at 16 [3], others at 18–19 years of age [3, 17], and there are those who consider patients as old as 24 years to be young adults/adolescents that require specialized emergency care [17].

Further to defining the pediatric patient, it should be decided who would be the most appropriate care manager for pediatric trauma patients. The shortage in pediatric trauma professionals has led to situations where pediatric casualties are often treated by adult trauma physicians [18]. Indeed some studies have suggested that using adult general surgeons to care for pediatric patients may not result in optimal care given the lower number of pediatric cases being performed by general surgery trainees [19]. Although some of our interviewees thought that pediatric surgeons should be the case managers of pediatric trauma patients, most of them stated that management should reside with the pediatric emergency medicine expert. Both views can be supported by research findings. Pediatric surgeons have been credited with providing more accurate diagnoses and better long-term outcomes for pediatric trauma casualties [20, 21]. However, a larger body of research supports a leadership role for the pediatric emergency medicine specialist in the management of pediatric trauma care, using the pediatric surgeon as an ad-hoc consultant [22–25]. This may be further supported by the shortage of pediatric surgeons in hospitals, as mentioned by the interviewees; they are often occupied in the operating rooms and are not available for treating trauma cases.

One element that evoked consensus among all study participants is the shortage in qualified staff that can provide around-the-clock optimal emergency care to pediatric trauma casualties. This is in line with previous findings that described the human resource as the single most important factor for a successful pediatric emergency care system [26, 27]. Implementation of an effective trauma team and trauma service was associated with a significant reduction in delayed diagnosis of injury team [28], decreased time required for ED treatment of severe trauma and improved survival [29]. In a recent publication, the American Committee on Pediatric Emergency Medicine stated that the scarcity of pediatric trauma care specialists, especially those with critical care training, constitutes a significant risk to children that have been afflicted by traumatic injuries and severely impedes the delivery of quality care [1]. This shortage and its severe implications have been previously described [3, 30].

There was a disagreement among the participants regarding the optimal setting for providing the best and most successful care for children with traumatic injuries. Several participants considered this to be general EDs, particularly for pediatric casualties with major trauma, because their staff comprises emergency medicine specialists, while others considered pediatric EDs to be the best suited for admitting these patients upon arrival. Presented with the option of a designated pediatric ED, many of the participants perceived that specialized centers should be the destination for all major pediatric trauma cases, citing that such a center would have a very high level of expertise and be equipped with the most cutting-edge medical technology [26, 31]. It has been suggested that pediatric patients and the elderly who are at risk for higher mortality from their injuries are subpopulations that would most likely benefit from more aggressive care in designated trauma centers [32, 33]. Studies examining the optimal trauma center type for children have mostly shown an outcome advantage for children treated at a pediatric trauma center or mixed trauma center as compared to those treated at adult trauma centers [34–41]. Several studies have shown that children who were treated in pediatric trauma centers had higher success rates when treated conservatively [4, 42], shorter stays and consequently lower costs to the medical center [37, 43]. The Committee on Pediatric Emergency medicine recommended that the pediatric patient should be stabilized at the closest medical center and then transferred to a designated trauma center [1]. Controversial results on the effect of trauma center type on pediatric mortality were reported in several studies. Amini et al. [44] reported improved survival in children treated at pediatric trauma centers than those treated at all other levels of adult trauma centers. Potoka et al. [37] found improved survival for children treated at pediatric trauma centers and adult trauma centers with added qualifications in pediatrics than those treated at level I or level II adult trauma centers. Sherman et al. [45] reported improved survival for children treated at level I adult trauma centers and adult trauma centers with added qualifications in pediatrics when compared with those treated at pediatric trauma centers or level II adult trauma centers. Conversely, Osler et al. [46] did not find significant differences in pediatric trauma mortality between adult trauma centers and pediatric trauma centers.

Detractors of the designated pediatric trauma center approach claimed that it would prevent staff in other hospitals from experiencing pediatric trauma care and acquiring the skills needed for handling such cases. A solution for this is to have rotations of experts among EDs and designated pediatric EDs. This would enable medical teams to increase their exposure to pediatric trauma cases and to enhance and maintain their skills. Another layer of this discussion involved the physical location of the pediatric ED, i.e., whether severely injured children should be evacuated to a small number of centrally-located hospitals or whether such care should be dispersed over many geographical regions. Currently available research findings provide little in the way of concrete guidance on this matter, because of the difficulty of studying the centralization paradigm due to adjustments for case mix differences between specialty and non–specialty hospitals, a limited number of assessed outcomes, and failure to account for the quality of individual hospitals in the analysis.

Our participants were not unanimous regarding the type of training that is optimal for teams that treat pediatric trauma casualties. The prevailing notion among them was that the current level of training is sub-optimal and that training should be enhanced. In Israel there are no guidelines on the type of trauma training required by medical staff, and specifically nurses, who deal with trauma cases. Advanced trauma life support certification has become mandatory for all surgery residents [9]. As for nurses, the head nurse usually decides upon the type of training, which is commonly pediatric intensive care or pediatric emergency medicine. Similarly, the Committee on Pediatric Emergency Medicine of the American Academy of Pediatrics has found significant variability in pediatric training and experience among physicians and nurses working in EDs [1]. Researchers have determined that specialized training in pediatric emergency care is crucial for emergency medicine teams and that the initial training should be supplemented by periodic refresher courses. In fact, it was strongly recommended that specialized training be supported by renewable certifications [47].

Leaders in the field of pediatric trauma in Israel have long been aware that the current state of resource availability, management structure and personnel training in the field of emergency medicine are sub-optimal. This awareness has led to the formation of a subcommittee of the National Council on Emergency Medicine and Trauma in the Israeli Ministry of Health with the aim of issuing national-level recommendations for the improvement of management of pediatric trauma casualties in designated adult trauma centers, level II hospitals, and emergency medical systems in Israel. The committee was led by a pediatric emergency physician and, in addition, included: a trauma surgeon, a pediatric surgeon, a pediatric intensivist and hospital administrator, a pediatrician, the medical director of the ED and an additional pediatric emergency physician. The committee took into consideration the wide variability in pediatric trauma management among hospitals, and wrote, based on a comprehensive analysis of the Israeli national trauma registry, specific recommendations on the upper age cutoff, treatment site, team composition, infrastructure and equipment, educational and training programs as well as ways to preserve skills and knowledge acquired (Waisman Y, Kessel B, Herbertal M, et al.: Report of the task force on improving pediatric trauma systems of the National Council on Emergency Medicine and Trauma. Waisman Y, personal communication of unpublished data). These recommendations will be developed into a model of pediatric trauma causalities’ intake and care to be approved as guidelines by the Ministry of Health’s directive with the hope of improving outcomes and reducing disability of injured children.

One of the limitations of the study is that the views represented are specific to its 17 participants; however we believe that their positions as leaders in their fields make them appropriate representatives of their sectors. In addition, interpretation by reflection of responses was conducted by the authors through their professional and own opinions. Nonetheless, the researchers attempted to minimize the potential effect of the investigator’s personal biography by maintaining an investigator’s diary in which thoughts, feelings and subjective experiences that were raised during the study were listed, and by review of the interviews and analyses by an independent colleague in order to assist the preservation of the original context as given in the interviews. Further research will examine the views and perspectives of emergency medicine teams (physicians and nurses) working in EDs across Israel.

## Conclusions

Our findings indicate that specialized pediatric EDs, located at a defined number of hospitals would constitute the best location for intake of children with major traumatic injuries. Pediatric emergency medicine specialists should manage trauma cases using pediatric surgeons as ad-hoc consultants. The term ‘pediatric patient’ should be defined to allow trauma patients to be referred to the most appropriate ED. Teams working at these EDs should undergo specialized pediatric emergency medicine training. Finally, to regulate the key aspects of trauma care, clear statutory guidelines should be formulated at the national and local levels.

## References

[1] Committee on Pediatric Emergency Medicine, Council on Injury Violence and Poison Prevention, Section on Critical Care, Section on Orthopaedics, Section on Surgery, Section on Transport Medicine, Pediatric Trauma Society, Society of Trauma Nurses Pediatric Committee. Management of Pediatric Trauma. Pediatrics 2016;138. 10.1542/peds.2016-1569.

[2] Flynn-O'Brien KT, Thompson LL, Gall CM, Fallat ME, Rice TB, Rivara FP (2016). Variability in the structure and care processes for critically injured children: a multicenter survey of trauma bay and intensive care units. J Pediatr Surg.

[3] Gausche-Hill M, Schmitz C, Lewis RJ (2007). Pediatric preparedness of US emergency departments: a 2003 survey. Pediatrics.

[4] Ochoa C, Chokshi N, Upperman JS, Jurkovich GJ, Ford HR (2007). Prior studies comparing outcomes from trauma care at children's hospitals versus adult hospitals. J Trauma.

[5] Negus S, Danin J, Fisher R, Johnson K, Landes C, Somers J, Fitzsimmons C, Ashford N, Foster J (2014). Paediatric trauma imaging: why do we need separate guidance?. Clin Radiol.

[6] Stone KP, Woodward GA (2010). Pediatric patients in the adult trauma bay - comfort level and challenges. Clin Pediatr Emerg Med.

[7] MacKenzie EJ, Rivara FP, Jurkovich GJ, Nathens AB, Frey KP, Egleston BL, Salkever DS, Scharfstein DO (2006). A national evaluation of the effect of trauma-center care on mortality. N Engl J Med.

[8] Remick K, Kaji AH, Olson L, Ely M, Schmuhl P, McGrath N, Edgerton E, Gausche-Hill M (2016). Pediatric readiness and facility verification. Ann Emerg Med.

[9] Peleg K, Aharonson-Daniel L, Stein M, Kluger Y, Michaelson M, Rivkind A, Boyko V, Israel TG (2004). Increased survival among severe trauma patients: the impact of a national trauma system. Arch Surg.

[10] Waisman Y. Establishing pediatric emergency medicine in Israel: reflections and lessons. Clin Pediatr Emerg Med. 2012;13:18–24.

[11] Lederman Z, Wacht O (2014). Family presence during resuscitation: attitudes of Yale-new haven hospital staff. Yale J Biol Med.

[12] Shkedi A (2014). Words of meaning: qualitative research-theory and practice.

[13] Elo S, Kääriäinen M, Kanste O, Pölkki T, Utriainen K, Kyngäs H. Qualitative content analysis: a focus on trustworthiness. SAGE Open. 2014:1–10.

[14] Committee on Trauma American College of Surgeons (2014). Resources for optimal care of the injured patient.

[15] Ayatollahi H, Bath PA, Goodacre S, Lo SY, Draegebo M, Khan FA (2013). What factors influence emergency department staff attitudes towards using information technology?. Emerg Med J.

[16] Waisman Y, Amir L, Mor M, Feigenberg Z, Daniel Aharonson L, Peleg K, Blumenfeld A (2006). Prehospital response and field triage in pediatric mass casualty incidents: the israeli experience. Clin Pediatr Emerg Med.

[17] Elbarbary M, Hancock BJ, Morris MI. Trauma in the pediatric patient. Trauma Team Dynamics. 2016;133

[18] Remick K, Snow S, Gausche-Hill M (2013). Emergency department readiness for pediatric illness and injury. Pediatr Emerg Med Pract.

[19] Gow KW, Drake FT, Aarabi S, Waldhausen JH (2013). The ACGME case log: general surgery resident experience in pediatric surgery. J Pediatr Surg.

[20] Berg BM, Salzman GA, Burke RV, Santore MT, Dingeldein MW, Upperman JS (2016). The pediatric surgeon's readiness to respond: commitment to advance preparation and effective coordinated response. J Pediatr Surg.

[21] Leeper WR, Leeper TJ, Vogt KN, Charyk-Stewart T, Gray DK, Parry NG (2013). The role of trauma team leaders in missed injuries: does specialty matter?. J Trauma Acute Care Surg.

[22] Crosby BJ, Mannelli F, Nisavic M, Passannante A, Cline DM, Gillespie CP, Messineo A, Ban KM (2013). The impact of implementing the single provider model of emergency medicine in a paediatric hospital: a retrospective cohort analysis. Emerg Med J.

[23] Gausche-Hill M, Johnson RW, Warden CR, Brennan JA, American College of Emergency Physicians’ Pediatric Emergency C (2003). The role of the emergency physician in emergency medical services for children. Ann Emerg Med.

[24] Green SM (2011). Trauma is occasionally a surgical disease: how can we best predict when?. Ann Emerg Med.

[25] Vernon DD, Bolte RG, Scaife E, Hansen KW (2005). Alternative model for a pediatric trauma center: efficient use of physician manpower at a freestanding children's hospital. Pediatr Emerg Care.

[26] Kim DK (2011). Regionalization of pediatric emergency care in Korea. Korean J Pediatr.

[27] Gausche-Hill M, Ely M, Schmuhl P, Telford R, Remick KE, Edgerton EA, Olson LM (2015). A national assessment of pediatric readiness of emergency departments. JAMA Pediatr.

[28] American Academy of Pediatrics Section on Orthopaedics, American Academy of Pediatrics Committee on Pediatric Emergency Medicine, American Academy of Pediatrics Section on Critical Care, American Academy of Pediatrics Section on Surgery, American Academy of Pediatrics Section on Transport Medicine, American Academy of Pediatrics Committee on Pediatric Emergency Medicine, Pediatric Orthopaedic Society of North America, Krug SE, Tuggle DW. Management of pediatric trauma. Pediatrics 2008;121:849-854.

[29] Vernon DD, Furnival RA, Hansen KW, Diller EM, Bolte RG, Johnson DG, Dean JM (1999). Effect of a pediatric trauma response team on emergency department treatment time and mortality of pediatric trauma victims. Pediatrics.

[30] Perno JF, Schunk JE, Hansen KW, Furnival RA (2005). Significant reduction in delayed diagnosis of injury with implementation of a pediatric trauma service. Pediatr Emerg Care.

[31] Lorch SA, Myers S, Carr B (2010). The regionalization of pediatric health care. Pediatrics.

[32] Grossman MD, Yelon JA, Szydiak L (2017). Effect of American College of Surgeons trauma center designation on outcomes: measurable benefit at the extremes of age and injury. J Am Coll Surg.

[33] Hsia RY, Wang E, Saynina O, Wise P, Perez-Stable EJ, Auerbach A (2011). Factors associated with trauma center use for elderly patients with trauma: a statewide analysis, 1999-2008. Arch Surg.

[34] Hall JR, Reyes HM, Meller JL, Loeff DS, Dembek R (1996). The outcome for children with blunt trauma is best at a pediatric trauma center. J Pediatr Surg.

[35] Potoka DA, Schall LC, Gardner MJ, Stafford PW, Peitzman AB, Ford HR (2000). Impact of pediatric trauma centers on mortality in a statewide system. J Trauma.

[36] Potoka DA, Schall LC, Ford HR (2001). Improved functional outcome for severely injured children treated at pediatric trauma centers. J Trauma.

[37] Densmore JC, Lim HJ, Oldham KT, Guice KS (2006). Outcomes and delivery of care in pediatric injury. J Pediatr Surg.

[38] Pracht EE, Tepas JJ, Langland-Orban B, Simpson L, Pieper P, Flint LM (2008). Do pediatric patients with trauma in Florida have reduced mortality rates when treated in designated trauma centers?. J Pediatr Surg.

[39] Oyetunji TA, Haider AH, Downing SR, Bolorunduro OB, Efron DT, Haut ER, Chang DC, Cornwell EE, Abdullah F, Siram SM (2011). Treatment outcomes of injured children at adult level 1 trauma centers: are there benefits from added specialized care?. Am J Surg.

[40] Sathya C, Alali AS, Wales PW, Scales DC, Karanicolas PJ, Burd RS, Nance ML, Xiong W, Nathens AB (2015). Mortality among injured children treated at different trauma center types. JAMA Surg.

[41] Mitchell RJ, Curtis K, Chong S, Holland AJ, Soundappan SV, Wilson KL, Cass DT (2013). Comparative analysis of trends in paediatric trauma outcomes in new South Wales, Australia. Injury.

[42] Stylianos S, Nathens AB (2007). Comparing processes of pediatric trauma care at children's hospitals versus adult hospitals. J Trauma.

[43] Jen HC, Tillou A, Cryer HG, Shew SB (2010). Disparity in management and long-term outcomes of pediatric splenic injury in California. Ann Surg.

[44] Amini R, Lavoie A, Moore L, Sirois MJ, Emond M (2011). Pediatric trauma mortality by type of designated hospital in a mature inclusive trauma system. J Emerg Trauma Shock.

[45] Sherman HF, Landry VL, Jones LM (2001). Should level I trauma centers be rated NC-17?. J Trauma.

[46] Osler TM, Vane DW, Tepas JJ, Rogers FB, Shackford SR, Badger GJ (2001). Do pediatric trauma centers have better survival rates than adult trauma centers? An examination of the National Pediatric Trauma Registry. J Trauma.

[47] Eppich WJ, Brannen M, Hunt EA (2008). Team training: implications for emergency and critical care pediatrics. Curr Opin Pediatr.

